# Evaluation of *in vitro* Assays to Assess the Modulation of Dendritic Cells Functions by Therapeutic Antibodies and Aggregates

**DOI:** 10.3389/fimmu.2019.00601

**Published:** 2019-03-28

**Authors:** Hannah Morgan, Su-Yi Tseng, Yann Gallais, Margret Leineweber, Pascale Buchmann, Sabrina Riccardi, Myriam Nabhan, Jeannette Lo, Zaahira Gani, Natacha Szely, Cornelia S. Zhu, Ming Yang, Andrea Kiessling, Hans-Werner Vohr, Marc Pallardy, Fred Aswad, Isabelle Turbica

**Affiliations:** ^1^Translational Immunology, Discovery & Investigative Safety, Preclinical Safety, Novartis Institute for Biomedical Research, Basel, Switzerland; ^2^Biologics Research, Lead Discovery, Immunoprofiling, Bayer US LLC, San Francisco, CA, United States; ^3^Inflammation, Chimiokines et Immunopathologie, INSERM, Fac. de pharmacie - Univ. Paris-Sud, Université Paris-Saclay, Châtenay-Malabry, France; ^4^Immunotoxicology, Pharmaceuticals, Research and Development, Bayer AG, Wuppertal, Germany

**Keywords:** anti-drug antibodies, immunogenicity, dendritic cells, *in vitro* assays, aggregates, intracellular signaling

## Abstract

Therapeutic antibodies have the potential to induce immunogenicity leading to the development of anti-drug antibodies (ADA) that consequently may result in reduced serum drug concentrations, a loss of efficacy or potential hypersensitivity reactions. Among other factors, aggregated antibodies have been suggested to promote immunogenicity, thus enhancing ADA production. Dendritic cells (DC) are the most efficient antigen-presenting cell population and are crucial for the initiation of T cell responses and the subsequent generation of an adaptive immune response. This work focuses on the development of predictive *in vitro* assays that can monitor DC maturation, in order to determine whether drug products have direct DC stimulatory capabilities. To this end, four independent laboratories aligned a common protocol to differentiate human monocyte-derived DC (moDC) that were treated with either native or aggregated preparations of infliximab, natalizumab, adalimumab, or rituximab. These drug products were subjected to different forms of physical stress, heat and shear, resulting in aggregation and the formation of subvisible particles. Each partner developed and optimized assays to monitor diverse end-points of moDC maturation: measuring the upregulation of DC activation markers via flow cytometry, analyzing cytokine, and chemokine production via mRNA and protein quantification and identifying cell signaling pathways via quantification of protein phosphorylation. These study results indicated that infliximab, with the highest propensity to form aggregates when heat-stressed, induced a marked activation of moDC as measured by an increase in CD83 and CD86 surface expression, IL-1β, IL-6, IL-8, IL-12, TNFα, CCL3, and CCL4 transcript upregulation and release of respective proteins, and phosphorylation of the intracellular signaling proteins Syk, ERK1/2, and Akt. In contrast, natalizumab, which does not aggregate under these stress conditions, induced no DC activation in any assay system, whereas adalimumab or rituximab aggregates induced only slight parameter variation. Importantly, the data generated in the different assay systems by each partner site correlated and supported the use of these assays to monitor drug-intrinsic propensities to drive maturation of DC. This moDC assay is also a valuable tool as an *in vitro* model to assess the intracellular mechanisms that drive DC activation by aggregated therapeutic proteins.

## Introduction

The clinical use of therapeutic antibodies has enabled significant improvements in the treatment of an increasing number of severe diseases. However, all biopharmaceuticals have immunogenic potential in patients, leading to the development of anti-drug antibodies (ADA) that may have neutralizing effects on the drug, resulting in reduced effective concentrations of the therapeutic biopharmaceutical in serum, and a potentially reduced clinical response (1, 2). ADA may also induce potential hypersensitivity reactions and adverse effects such as infusion reactions (3–5). Immunogenicity of therapeutic antibodies has been particularly studied in the context of inflammatory diseases. Indeed, ADA development in patients has been reported with variable frequencies, depending on clinical studies that include different patient populations, as well as on the employed detection method. Although the humanization status of the administrated antibody allows for a potential reduction in immunogenicity, it is rarely abolished. Thus, ADA frequencies against chimeric antibodies such as infliximab or rituximab may vary from 10 to 50% (6–8), whereas ADA frequencies in patients treated with the fully human antibody adalimumab may range from 20 to 25% (6, 9, 10) and patients treated with the humanized antibody natalizumab may develop ADA with a frequency of ~6–10% (11, 12).

A variety of patient-specific and bioproduct-specific factors are involved in the onset and progression of immunogenicity (13–15). Among factors related to the drug product itself, it is now well-accepted that protein aggregation is associated with an increased potential for immunogenicity (16, 17). The aggregation process of therapeutic proteins may occur at any stage of the manufacturing process, storage, transport, or delivery to the patient. It is governed by a variety of critical environmental conditions (e.g., temperature, pH, ionic strength, shear forces) that can alter the protein either by physical or chemical damage (18, 19) and trigger the protein aggregation through different pathways. Thus, aggregation can occur either between unfolded antibody monomers or between folded molecules (20). In clinical practice, the use of concentrated antibody preparations that are administrated subcutaneously may favor the aggregation process because of the forced interaction of monomers in a confined space (21). Also, micro- and nanoparticles have been detected in the solutions prepared for intravenous administration (22).

Interactions of protein aggregates with the immune system have been studied through *in vivo* models that in particular highlighted a correlation between antibody aggregates and ADA development (23–26). Furthermore, *in vitro* studies focused on aggregate interactions with immune cells, in order to address the mechanisms leading to ADA production. Two hypotheses are currently explored, well-described by Moussa et al. (27). One suggests that aggregates can bind to B-cell receptors through repetitive conformational epitopes and elicit T-cell independent polyclonal B-cell activation with low affinity ADA production. The second hypothesis is that aggregates are recognized and captured more efficiently by antigen presenting cells (APC) and elicit a T-cell dependent activation of B-cells that undergo isotype switch to produce high affinity ADA. In this latter context, dendritic cells (DC) are the most efficient APC population that are crucial for the initiation of T cell responses and the subsequent generation of immunogenicity. Thus, DC can potentially interact with therapeutic antibodies and aggregates through innate receptors such as immunoglobulin receptors (FcγRs), danger signal receptors (e.g., TLRs), lectin receptors, or complement receptors as these molecules are broadly expressed on DC surface (28–30). Indeed it was shown that IgG1 aggregates have a high affinity for purified FcγR (31). Another study highlighted that TLR2 and TLR4, as well as FcγRI and FcγRIII, and the C3b protein were involved in the secretion of inflammatory cytokines by PBMC stimulated by stir-stressed monoclonal antibodies (32).

Recent work has provided some evidence that DC could be a valuable model to assess the potential immunogenicity of therapeutic antibodies and their aggregates. Indeed it was demonstrated that IgG aggregates induced monocyte-derived dendritic cell (moDC) maturation with the upregulation of phenotypic maturation markers and production of inflammatory cytokines (33–35). Moreover, peptides presented by HLA-DR molecules were identified and it was shown that highly aggregated antibodies increased the quantity of antigen-derived HLA-DR associated peptides (33). Also, uptake of IgG aggregates and localization in moDC endosomal compartment was increased, compared to monomeric counterparts (34). Finally the activation level of moDC stimulated with aggregated rituximab or aggregated polyclonal IgG were sufficient to promote CD4+ T-cell proliferation and cytokine secretion (35).

This study focuses on optimizing methods to monitor diverse end-points of DC activation by therapeutic antibodies and aggregates. Our goal is to define the appropriate read outs and settings to evaluate moDC maturation, by developing predictive *in vitro* assays that determine whether antibody preparations have intrinsic DC stimulatory capabilities that are independent of the antigen recognition (Fab) part of the molecule. For this purpose, four assays were designed to test the effect of four therapeutic monoclonal antibodies (infliximab, rituximab, adalimumab and natalizumab) currently used for treating inflammatory diseases, and to test the related aggregated preparations, on moDC cultures. Each assay was independently evaluated by four different laboratories for at least 12 donors.

## Materials and Methods

### Preparation of Antibody Aggregates

Antibodies used for this study were commercially available infliximab (Remicade®, 10 mg/mL), a chimeric anti-TNF-α antibody, rituximab (Mabthera®, 10 mg/mL), a chimeric anti-CD20 antibody, natalizumab (Tysabri®, 20 mg/mL), a humanized antibody which binds to α4β1-integrin and adalimumab (Humira®, 50 mg/mL), which is a fully human anti-TNF-α antibody. Antibodies preparations were subjected to either shear or heat stress, with two different intensities. Shear stresses was induced by filling and emptying a syringe through a 19 G needle for three (shear stress level 1, SSL1) or 10 (shear stress level 2, SSL2) cycles. Heat stress consisted of heating the samples at 55°C for 6 h (heat stress level 1, HSL1) or 24 h (heat stress level 2, HSL2). Aggregates were characterized by measuring particle numbers by microflow imaging, dynamic light scattering, size exclusion chromatography, and turbidity analysis. Detailed description of aggregates preparation and characterization is described elsewhere (manuscript in preparation). Native or aggregated antibodies were prepared and aliquoted by a single laboratory partner and conserved at −80°C. Aliquots were distributed among the other partners and kept at −80°C until use.

### Generation of Human Monocyte-Derived Dendritic Cells (moDC)

Human peripheral blood mononuclear cells (PBMCs) were purified from buffy coats or whole blood directly obtained from Blood donation centers: Etablissement Français du Sang (France), Interregionale Blutspende Schweizerisches Rotes Kreuz (Switzerland) and CRS Clinical Research Services, Wuppertal (Germany). For each site, healthy donors gave their written consent for the use of blood donation for research purposes. Also, PBMC directly isolated and frozen were purchased from vendor (AllCells, CA, USA).

In each partner lab's, PBMC isolation was achieved by density gradient centrifugation. Monocytes were isolated from the mononuclear fraction either by magnetic positive selection with MiniMacs or MidiMacs separation columns and anti-CD14 antibodies coated on magnetic beads (Miltenyi Biotech). Purified monocytes were cultured in the presence of GM-CSF (50 ng/ml) and IL-4 (50 ng/ml) (R&D Systems) in RPMI-1640 medium supplemented with 10% fetal calf serum (FCS), 1% Penicillin/Streptomycin, 1% Pyruvate, 1% L-glutamine, 1% HEPES, and 1% non-essential amino acids, at 37°C in humidified air containing 5% CO2. Cytokines were replenished in the cell culture on day 3 and within 6 days monocytes had differentiated into moDCs with an immature phenotype (CD11c+/CD14–). On Day 6, a quality control of the immature DC population was performed by flow cytometry.

### moDC Treatment With Aggregated or Native Antibodies

On Day 6, immature moDC were harvested and washed in RPMI-1640 medium supplemented with 10% FCS, 1% Penicillin/Streptomycin and 1% Pyruvate. Cells were counted in the presence of trypan blue to confirm viability. Following centrifugation (5 min, 360 g, 4°C) supernatants were discarded and cells were resuspended in warm medium and then seeded in the appropriate plates, depending on the experiment. Aggregates or native antibodies were then added at concentrations of 10, or 100 μg/mL. Maturation cocktail (IL-6, IL-1β, TNF-α, at 10 ng/mL, and PGE-2 at 1 μg/mL, all from R&D Systems or Sigma-Aldrich) or Lipopolysaccharide (LPS, from Escherichia coli 055:B5 strain 25 ng/ml, Sigma-Aldrich) were used as positive maturation controls. The stimulation time at 37°C ranged from 15 min to 48 h depending on the experiment.

### Flow Cytometric Analysis

Immature moDC were seeded into 24-well cell culture plates (Nunc, Langenselbold, Germany) at 1 mL per well (1 × 10^6^/well). 50 μL of test items were added to each well to reach final concentrations of test items 10 or 100 μg/mL. Cells were incubated with test items at 37°C and 5% CO2 for 48 h. All conditions were tested in singlicates as low inter-well variability was previously demonstrated for the method.

Multicolour immunofluorescence was performed using the following panel of mouse anti- human monoclonal antibodies: PE-Cy5 conjugated anti-CD11c (clone B-ly6), FITC conjugated CD80 (clone L307.4), PE-Cy7 anti human CD83 (clone HB15e), PE conjugated CD86 (clone FUN-1) (all from Becton Dickinson) in combination with a viability dye (LIVE/DEAD fixable dead cell stain RED; Molecular Probes, Life Technologies). Negative controls were isotype matched control antibodies (Becton Dickinson).

Stimulated DCs were harvested by gentle scraping in the presence of cell dissociation buffer (Gibco, Life Technologies), washed in FACS buffer (PBS, 2% FCS, 2 mM EDTA and 0.05% Sodium Azide) and subsequently blocked in 100% FCS. 2.5 × 10^5^ cells were stained with either the antibody or isotype control panel.

Following staining cells were resuspended in FACS buffer containing fixative [0.8% formaldehyde (Beckman Coulter)] for flow cytometry analysis.

Flow cytometry measurements were performed on an FC500MPL flow cytometer (Beckman Coulter). Cell debris were eliminated from the analysis by forward and side scatter gating and 10,000 viable CD11c+ cells were acquired using the MXP software (Becton Dickenson). Flow cytometry data for CD86 (MFI), CD80 (MFI), and CD83 (% positive cells) was analyzed using Kaluza Software (Becton Dickenson). Data are presented as the fold change over PBS treated control.

### Quantitative Real Time RT-PCR Assay (qPCR) of Cytokines and Chemokines

Immature moDC were plated in 24-well cell culture plates at 1 mL per well (1 × 10^6^/well) and then stimulated with 10 or 100 μg/mL of native or aggregated antibodies for 6 or 24 h. Total RNA was extracted after lysis using the Nucleospin RNA kit (Macherey Nagel, Hoerdt, France), according to the manufacturer's instructions. Total RNA pellets were resuspended in RNase-free water (60 μL) and quantified by spectrophotometry. First-strand cDNA was synthesized from total RNA by use of a thermocycler (Biometra, Gottingen, Germany). The reaction used 1 μg total RNA, a deoxynucleotide triphosphate mixture (containing 25 mM deoxy-adenosine triphosphate, deoxy-guanosine triphosphate, deoxy-cytidine triphosphate, and deoxy-thymidine triphosphate), and 50 mM oligo (dT) primers (MWG Biotech, Ebersberg, Germany). Reverse transcription was carried out in 1X AMV RT reaction buffer (Promega, Charbonnières-les-Bains, France) with RNase inhibitor (RNasine; Promega) at 40 U/μl, AMV RT (Promega) at 10 U/μl, and RNase-free water to a final volume of 10 μl. A control without RT was used to confirm the absence of DNA contamination. Real-time PCR was performed by use of the SYBR Green technology on a CFX96 system (Bio-Rad Laboratories, Marnes la Coquette, France). Each reaction mix consisted of 1:20 diluted cDNA in 4 μl nuclease-free water; 0.5 mM each forward and reverse primer for IL-1β, IL-6, IL-8, IL-12p40, TNF-α, CCL2, CCL3, CCL4, CCL5, CXCL10, GAPDH, β-actin (Table 1); and SsoAdvanced Supermix (Bio-Rad Laboratories) in a total reaction volume of 10 μl. After 30 s at 95°C for Sso7d-fusion polymerase activation, amplification was allowed to proceed for 44 cycles, each consisting of denaturation at 95°C for 5 s and annealing/extension at 62°C for 5 s. 7 fold serial dilutions of mixed cDNA (from different samples) were analyzed for each target gene, allowing us to construct linear standard curves from which the efficiency of each PCR run was evaluated. SYBR Green fluorescence was detected at the end of each elongation cycle, after which a melting curve was constructed to confirm the specificity of the PCR products. Quantification was performed with Bio-Rad Laboratories CFX Manager software, and data were analyzed with the ΔΔCt method. Ratios were calculated as the geometrical mean of (1 + E)^−ΔΔCt^ values, where E is the efficiency, and ΔΔCt is the target gene expression of treated cells compared with normal levels in untreated cells, with correction for the expression of the reference genes βactin and GAPDH. Results were expressed as the fold-factor induction [i.e., ratio of (1 + E)^−ΔΔCt^ of treated cells/(1 + E)^−ΔΔCt^ of untreated cells].

**Table 1:** Forward and Reverse primers for q-PCR tested cytokines and chemokines.

	**Forward (5′-3′)**	**Reverse (5′-3′)**
lL-1β	ACA GAC CTI CCA GGA GAA TG	GCA GTI CAG TGA TCG TAC AG
ll-6	TCA ATG AGG AGA CTI GCC TG	GAT GAG TIG TCA TGT CCT GC-3
ll-8	TCT CTI GGC AGC CTI CCA TGA	TGG GGT GGA AAG GTI TGG AG
ll-12p40	TGG AGT GCC AGG AGG ACA GT	TCT TGG GTG GGT CAG GTI TG
TNF-α	TCT TCT CGA ACC CCG AGT GA	CCT CTG ATG GCA CCA CCA G
CCl3	TCT GCA ACC AGT TCT CTG CAT CAC	ACT GGC TGC TCG TCT CAA AGT A
CCl4	CGC CTG CTG CTI TIC TIA CAC	GGT TIG GAA TAC CAC AGC TIG
CCl5	GCC CAC ATC AAG GAG TAT TIC TAC A	CGG TIC TTT CGG GTG ACA A
CXCllO	TCT AAG TGG CAT TCA AGG AGT ACC	AAA GAC CTI GGA TIA ACA GGT TGA
GAPDH	CAG CCT CAA GAT CAT CAG CA	TGT CGT CAT GAG TCC TIC CA

### Cytokine and Chemokine Quantification in Cell Culture Supernatants

Immature moDC were plated in 48-well flat bottom tissue culture plates (Falcon) with 400 μl per well (1 × 10^6^cells/ml) and then stimulated with 10 or 100 μg/mL of native or aggregated antibodies. After 48 h stimulation moDC culture supernatants were measured in duplicate for cytokines IL-1β, IL-2, IL-4, IL-6, IL-8, IL-10, IL-12p70, IL-13, TNF-α, IFN-γ, and chemokines CCL2 (MCP-1), CCL3 (MIP-1α), CCL-4 (MIP-1β), and CXCL10 (IP-10), using Meso Scale Discovery (MSD, Rockville, MA, USA) multiplex assay, following manufacturer's instructions.

### Detection of Phosphoproteins Signaling Pathways

Cell lysates were obtained from immature moDC (2 × 10^6^ cells/ml) plated at 100 μl /well in a 96 well round bottom plate treated with 100 μg/ml of native antibodies or aggregates for 15 min or 30 min. Treated cells were washed in cold PBS then lysed for 5 min on ice with ice-cold 1 × Cell Lysis Buffer (Cell Signaling Technology, USA) plus Halt Protease and Phosphatase inhibitor (Pierce Protein Biology). Cell debris were removed by micro-centrifugation at max speed for 10 min at 4°C. According to manufacturer's instructions, samples were added to customized phospho signaling kit (Bio-Rad Laboratories) containing microbeads with antibodies against human Akt (Ser^473^), ERK1/2 (Thr^202^/Tyr^204^, Thr^185^/Tyr^187^ and Syk (Tyr^352^) phosphoproteins at 50 μl/well in 96 well assay plate (Greiner Bio-One). Data were acquired using Bio-Plex 200 system (Bio-Rad Laboratories) according to the manufacturer's recommended settings for phosphoprotein acquisition. Data values were normalized against reference control GAPDH, and sample values were divided by value for immature DC treated with media alone to achieve fold difference over baseline. Data were collected from 12 donors.

### Statistical Analysis

Data were expressed as means ± SEM. Differences between groups were evaluated with the Mann-Whitney U test (Prism software, GraphPad, La Jolla, CA, USA). *P*-values below 0.05 were considered to denote statistical significance.

## Results

Native or aggregated antibodies were used to evaluate their capacity to induce maturation of immature moDCs from healthy donors. To this end, moDC differentiation procedures were harmonized across four independent laboratories (Switzerland, France Germany and USA), and the same preparations -either of native or aggregated antibodies- were tested.

**Figure 1:**
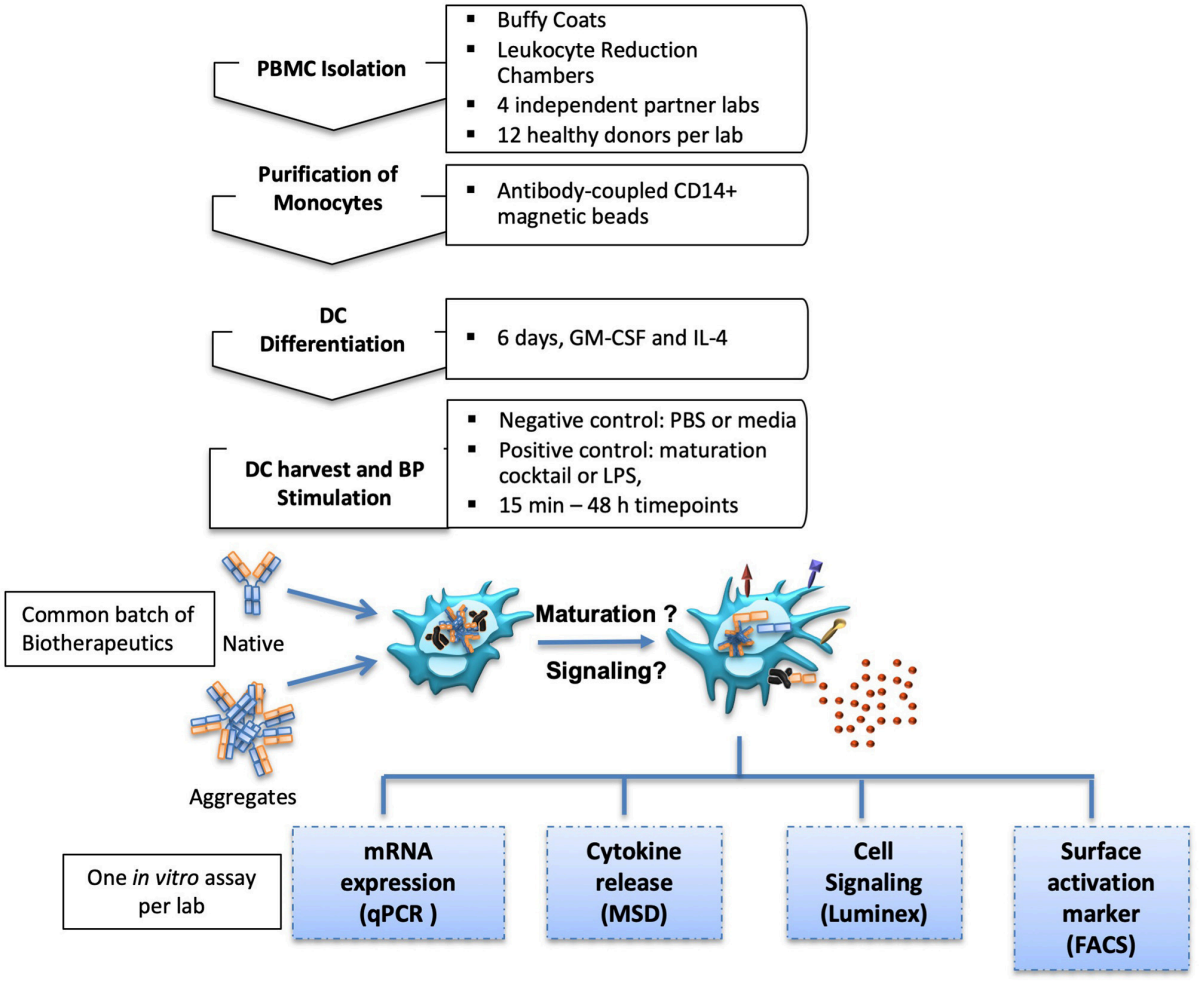
Experimental setup. MoDC isolation and differentiation protocols were aligned for four international partner laboratories. Generated moDC cultures were treated with native or aggregated forms of Abs. MoDC maturation was measured by different *in vitro* assays.

Four commercial antibodies (infliximab, rituximab, natalizumab, and adalimumab) were submitted to different stresses which could induce aggregates to be tested in the *in vitro* assays. Thus, shear stress and heat stress were tested, both with two increasing intensities, in order to assess potential dose-dependent effects of the formed particles. Antibody behavior under these stress conditions was rather different. Upon heat stress, infliximab and rituximab showed a fluffy aspect at the optical level, confirmed by a dramatic increase of the number of subvisible aggregates that was dependent on the stress intensity. Adalimumab and natalizumab preparations remained clear and showed only a slight increase of subvisible particles, independent of intensity (data not shown). On the other hand, infliximab and rituximab showed moderate susceptibility to shear stress, with less subvisible particles compared to heat stress. Natalizumab was also sensitive to shear stress but to a lesser extent, whereas adalimumab did not show any susceptibility to this stress. Full characterization of the aggregation of these antibodies is described elsewhere (manuscript in preparation). To study moDC maturation, each site performed one of the following *in vitro* assays: (1) analysis of maturation and co-stimulatory membrane protein expression by flow cytometry, (2) the expression of cytokine and chemokine mRNA measured by real time quantitative PCR, (3) quantification of secreted cytokine and chemokine secretion, and (4) analysis of intracellular phosphoproteins involved in DC signaling pathways (Figure 1).

### Phenotypic moDC Maturation Measured by Flow Cytometry

**Figure 2:**
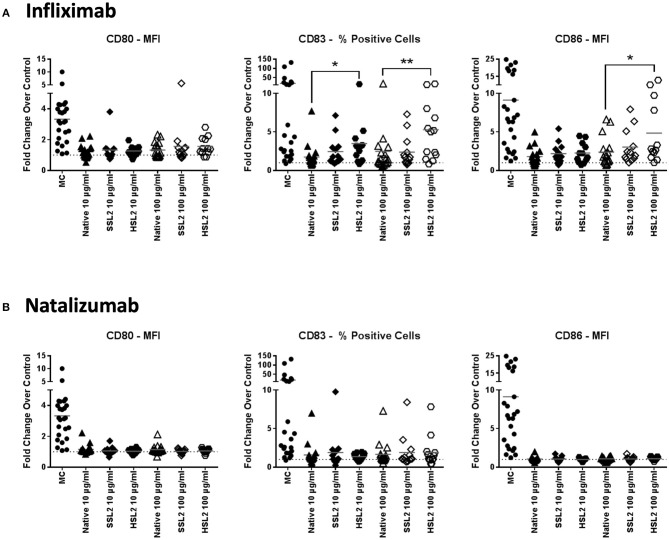
Phenotypic assessment of maturation marker expression on moDC following treatment with native or aggregated infliximab **(A)** and natalizumab **(B)**. Immature moDC were treated for 48 h with the positive control (MC; maturation cocktail as described in the Methods section), native or stressed (SSL2 or HSL2) therapeutic antibodies at 10 or 100 μg/mL. Cells were collected, washed, and analyzed by flow cytometry for CD80, CD83, and CD86 expression was measured on CD11c+ moDC. Results are expressed as the fold change of marker expression (either as the MFI or as the % of positive cells based on the sensitivity of responses to the positive control for each marker) compared to PBS treated cells (represented by the horizontal dotted line at 1.0). Results from 12–23 independent experiments are shown, individual points representing individual donor responses. The group's mean is represented by the horizontal gray line. *p < 0.05; **p < 0.01.

Immature moDC were incubated with 10 or 100 μg/mL of native or aggregated antibodies for 48 h. The upregulation of the maturation marker CD83 and the CD4 T-cell co-stimulatory molecules CD80 and CD86 relative to PBS treated cells was determined by flow cytometry via the evaluation of the mean fluorescence intensity (for CD86 and CD80), and the percentage of positive cells (for CD83). These parameters were selected as they showed the greatest response to the maturation cocktail positive control determined as the fold change over PBS treated cells for each marker, (e.g., MFI vs. % positive cells: CD86: 8.81x vs. 1.72x; CD80: 3.32x vs. 3.30x; CD83: 4.97x vs. 18.35x). The maturation cocktail positive control resulted in a significant upregulation of CD83, CD80, and CD86 in all tested donors when compared to the PBS treated control, however there was a significant inter-individual variability in this biological assay system (Figure 2). Treatment of immature moDCs with native antibodies induced only a slight increase in expression of all maturation markers, with fold changes over the PBS treated control remaining below 1.5 for all tested mAbs. Only infliximab treatment at 100 μg/mL increased both CD83 and CD86 expression (2.4 and 2.5-fold, respectively), although not statistically significant. No significant changes in surface marker expression were detected after treatment of immature moDCs with all SSL1 or SSL2 and HSL1 aggregated antibodies tested at any concentration (data for heat and shear stress level 1 not shown; SSL2 data in Figure 2 and Supplemental Figure 1). In contrast, a significant up-regulation of CD83 and CD86 was observed for HSL2 aggregated infliximab at 10 (CD83: *p* = 0.0160) and 100 μg/mL (CD83: *p* = 0.0085, and CD86: *p* = 0.0329; Figure 2A). Treatment of moDCs with all other HSL2 aggregated antibodies also led to no significant upregulation of maturation markers (Figure 2 and Supplemental Figure 1). This lack of a statistically significant response was likely due to the high level of donor variation in this assay system.

### Cytokine and Chemokine Transcripts Expression Measured by qPCR

**Figure 3:**
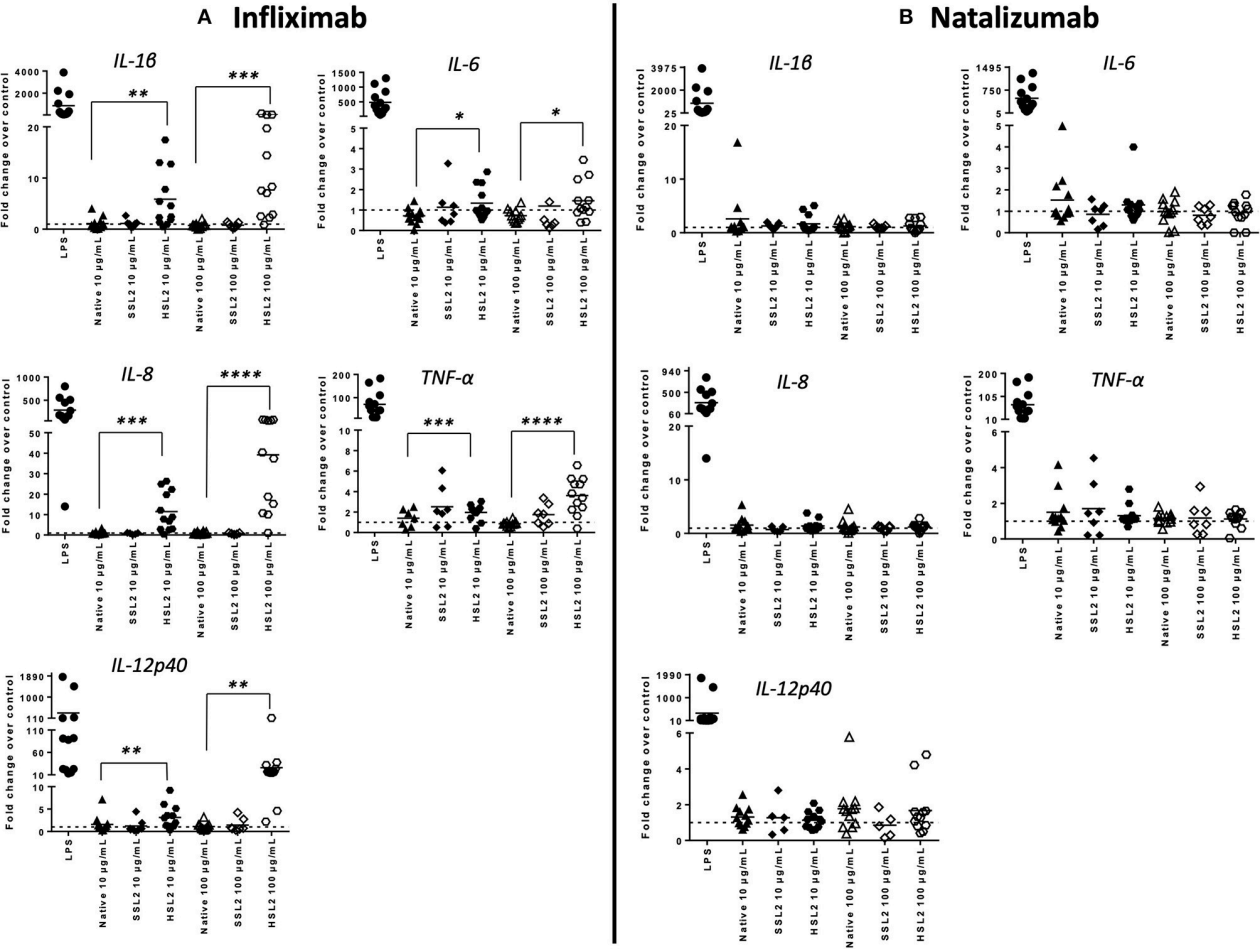
Expression of proinflammatory cytokine mRNA in moDC following treatment with native or aggregated infliximab **(A)** and natalizumab **(B)**. Immature moDC were treated with native or stressed (SSL2 or HSL2) antibodies. Transcripts were measured after 6 h (for IL-1β, IL-6, IL-8, and TNFα) or 24 h (for IL-12p40) using real-time RT-PCR. Results are expressed as fold change over PBS control. The results from 12 independent experiments are shown. *p < 0.05; **p < 0.01; ***p < 0.001; ****p < 0.0001.

**Figure 4:**
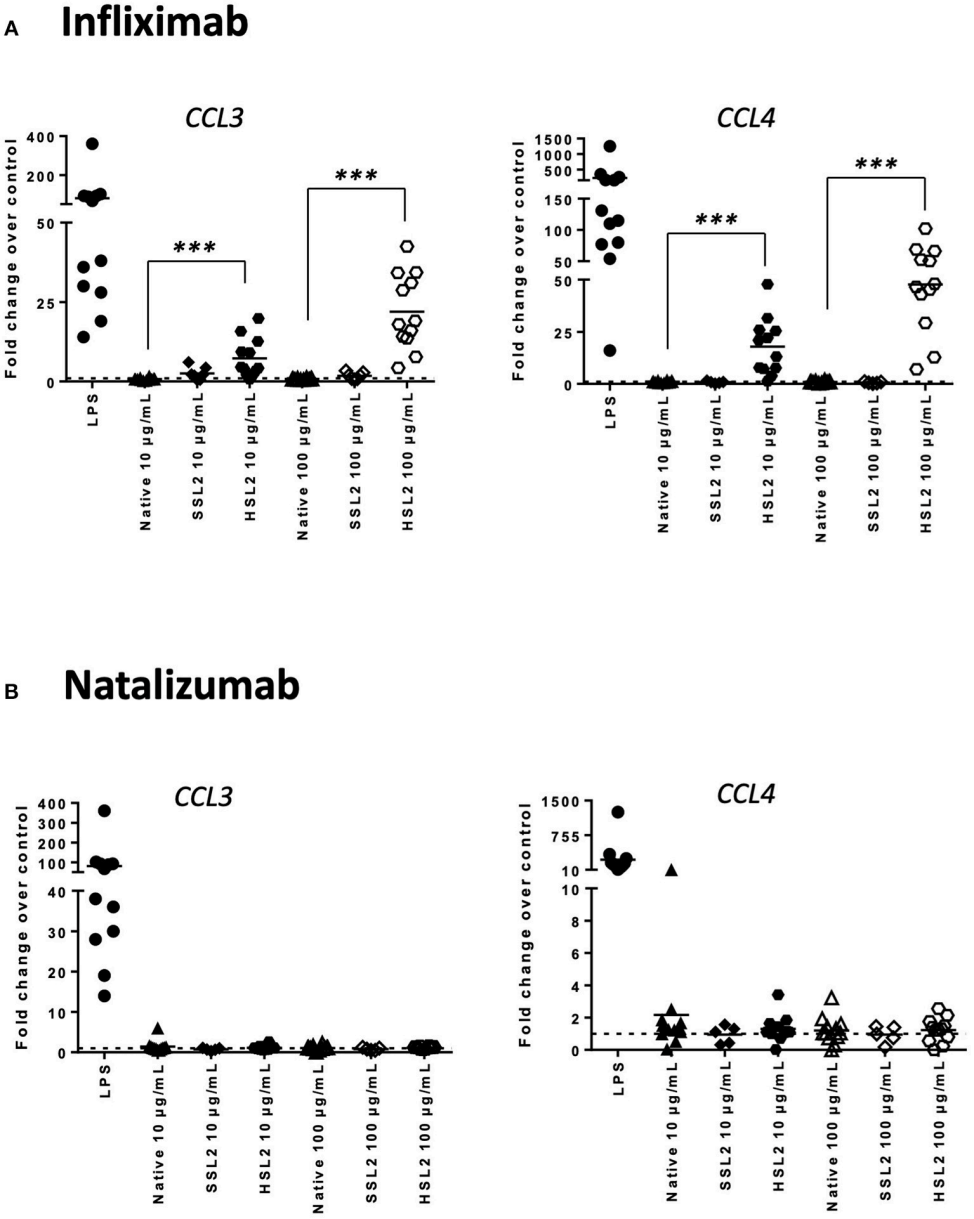
Expression of chemokines CCL3 and CCL4 mRNA in moDC treated with native or aggregated infliximab **(A)** and natalizumab **(B)**. Immature moDC were treated with native or stressed (SSL2 or HSL2) antibodies. Transcript expression was measured after 6 h using real-time RT-PCR. Results are expressed as fold change over PBS control. The results from 12 independent experiments are shown. ***p < 0.001.

MoDC maturation by antibodies and aggregates was also assessed by quantification of mRNA levels of several cytokines and chemokines, following 6 or 24 h of incubation. Native antibodies did not induce transcript upregulation. As expected, in the presence of LPS, the mRNA levels of all cytokines and chemokines were strongly up-regulated (Figure 3). Infliximab HSL2 aggregates induced a significant increase in IL-1β, IL-6, IL-8, and TNF-α, 6 h after stimulation, showing concentration dependency, whereas IL-12p40 mRNA was increased 24 h after stimulation (Figure 3). Infliximab HSL2 aggregates also up-regulated CCL3 and CCL4 transcripts (Figure 4). In contrast, other infliximab aggregates (SSL2, HSL1, and SSL1) did not induce any transcript upregulation (data not shown). For natalizumab (Figures 3, 4), adalimumab, and rituximab (data not shown), none of the stressed materials induced significant transcript upregulation.

### Quantification of Cytokines and Chemokines Release

**Figure 5:**
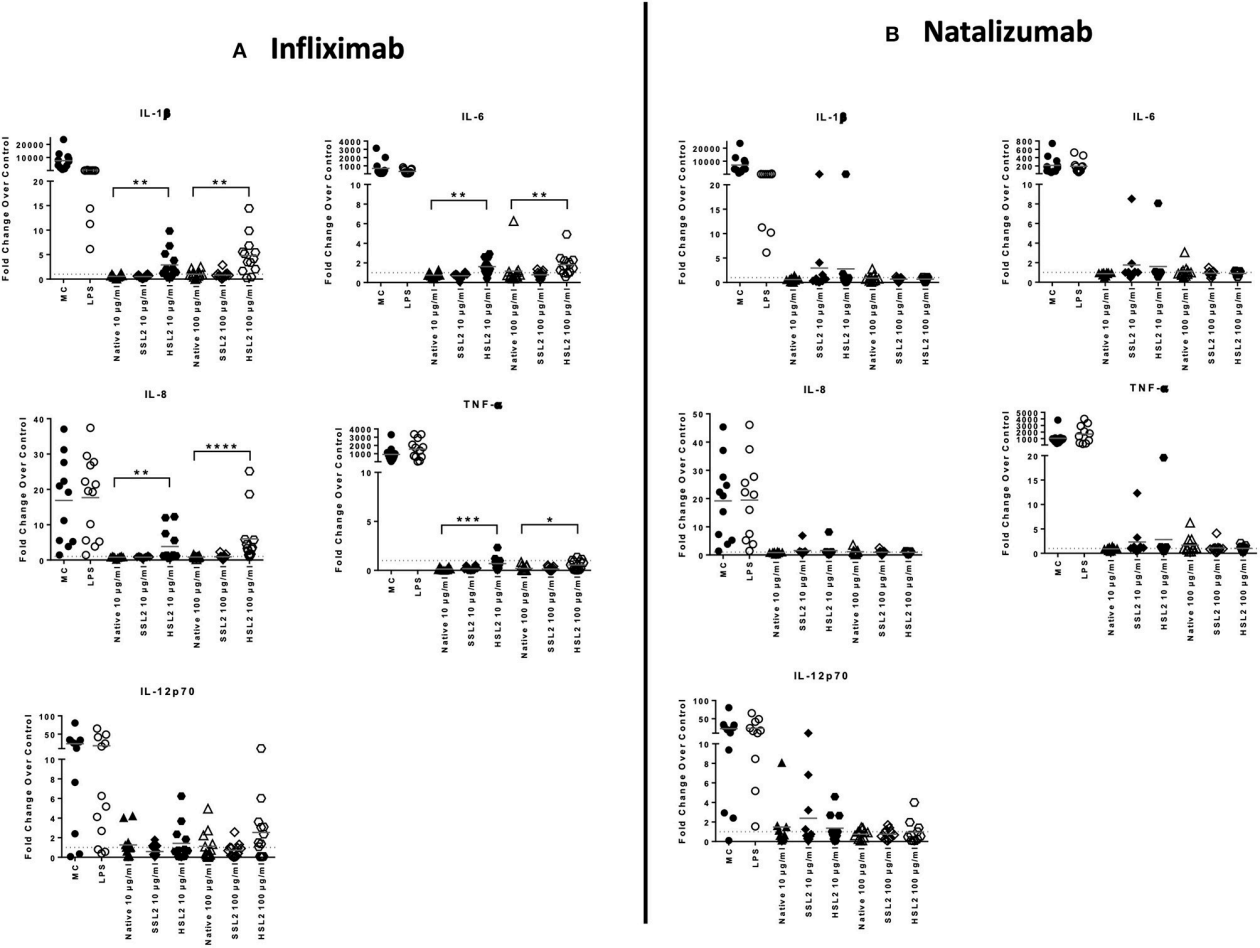
Protein levels of proinflammatory cytokines in moDC following treatment with native or aggregated infliximab **(A)** and natalizumab **(B)**. Immature moDC were treated for 48 h with native or stressed (SSL2 or HSL2) antibodies, or maturation cocktail (MC) or LPS. Cytokine concentrations were measured in culture supernatants using a MSD multiplex assay. Results are expressed as fold chage over PBS control. The results from 11 independent experiments are shown. *p < 0.05; **p < 0.01; ***p < 0.001; ****p < 0.0001.

**Figure 6:**
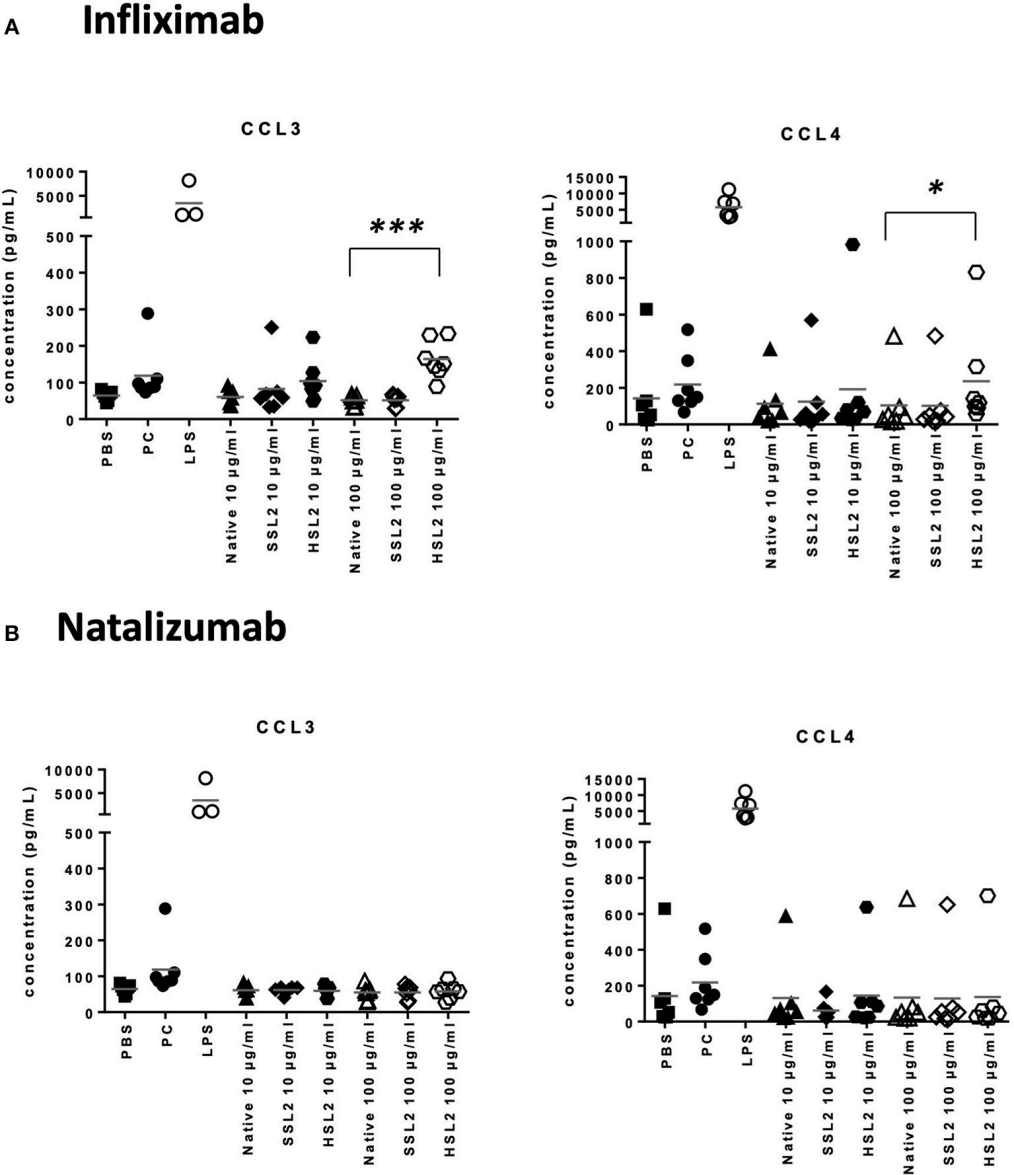
Secretion of chemokines CCL3 and CCL4 secretions by moDC treated with native or aggregated infliximab **(A)**, and natalizumab **(B)**. Immature moDC were treated for 48 h with native or stressed (SSL2 or HSL2) antibodies, maturation cocktail (MC) or LPS. Chemokine concentrations were measured in culture supernatants using a MSD multiplex assay. Results from seven independent experiments are shown. *p < 0.05; ***p < 0.001.

The production of cytokines and chemokines was measured 48 h after moDC stimulation by antibodies and aggregates, to see if changes in gene transcription could be correlated at the protein level. Positive controls (LPS or maturation cocktail described in Materials and Methods section) gave high levels of all cytokines production. After stimulation with infliximab HSL2 aggregates (10 and 100 μg/mL), significant increases of IL-1β, IL-6, IL-8, and TNF-α were observed compared to the native mAb (Figure 5A; data per donor are reported in Supplemental Table 1), that were consistent with the up-regulation of mRNA observed by q-PCR measurements. Moreover, there was a trend for IL-12p70 up-regulation (10 and 100 μg/mL), that can be correlated with the later increase of transcripts at 24 h after stimulation. Regarding chemokine production, infliximab HSL2 aggregates induced CCL3 and CCL4 up-regulation at 100 μg/mL (Figure 6A; data per donor are reported in Supplemental Table 3A), that was as well consistent with q-PCR measurements of mRNA counterparts. In contrast, other infliximab aggregates (SSL2, HSL1, and SSL1) did not induce any cytokine or chemokine production (data not shown). For natalizumab (Figures 5B, 6B; data per donor are reported in Supplemental Tables 2,3B), adalimumab, and rituximab (data not shown), none of the stressed materials induced significant secretions.

Taken together, our results showed that aggregated mAbs, in particular heat-stressed infliximab, increased cytokine, and chemokine production, both at the mRNA and protein level.

### Phosphoproteins Involved in moDC Signaling Pathways

**Figure 7:**
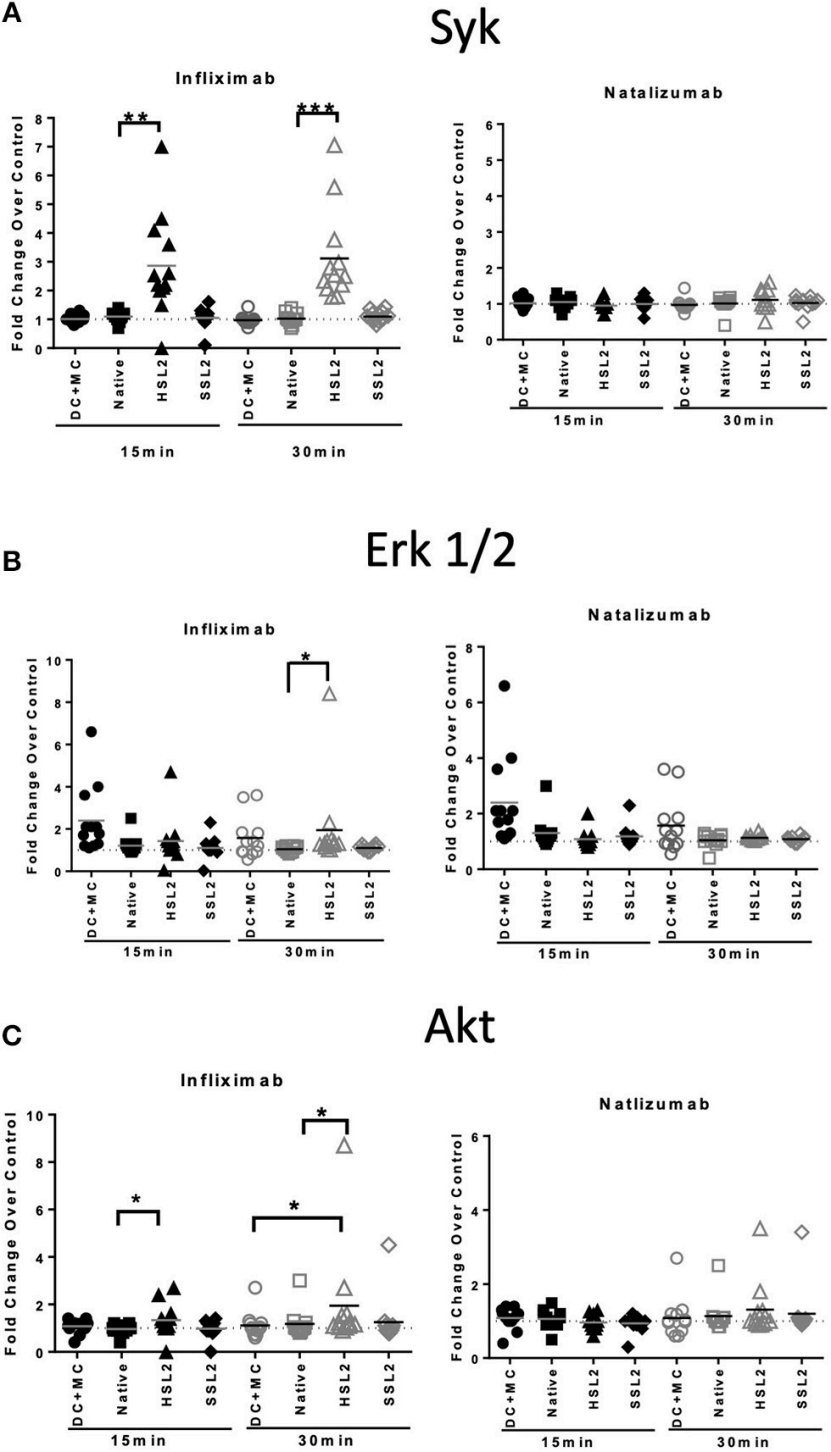
Protein phosphorylation in moDC treated with native or aggregated infliximab and natalizumab. **(A)** Syk phosphorylation, **(B)** ERK 1/2 phosphorylation, and **(C)** Akt phosphorylation. MoDC were treated with 100 μg/mL native or aggregated antibodies. Phosphorylation was detected following 15 and 30 min of stimulation. Results are expressed as fold change over PBS control. The results from 12 independent experiments are shown. *p < 0.05; **p < 0.01; ***p < 0.001.

In order to study the effect of mAbs and aggregates on phosphoproteins, we looked at the following signaling molecules that potentially play a role in DC activation: Akt (Ser^473^), ERK1/2 (Thr^202^/Tyr^204^, Thr^185^/Tyr^187^), and Syk (Tyr^352^). As expected, moDC treatment with the maturation cocktail resulted in all tested proteins phosphorylation except Syk. Our results show that heat-induced aggregates of infliximab (HSL2) significantly induced phosphorylation of Syk and Akt, at 15 and 30 min of treatment, and phosphorylation of ERK1/2, at 30 min of treatment (Figures 7A–C). The effect of infliximab on immature moDC seemed concentration dependent (Supplemental Figure 2). In contrast, no significant signaling activity was observed for natalizumab treatment (Figures 7A–C), neither for rituximab nor adalimumab treatments (Supplemental Figure 2).

## Discussion

The presence of aggregates in biotherapeutics has been correlated with ADA development in patients and many efforts are currently ongoing in an attempt to dissect the cellular mechanisms involved in immunogenicity. The aim of this study was to optimize *in vitro* methods to evaluate the potential of therapeutic antibodies and aggregated preparations on therapeutic antibodies to induce DC maturation, as these professional antigen-presenting cells have a pivotal role in triggering adaptive immune responses that would *in fine* lead to ADA production (27). Our approach was to test the impact of four therapeutic antibodies and aggregated formats created using physical stress using different assay methods performed by independent laboratories to establish robust readouts of dendritic cell maturation that could be used to monitor bioproduct preparations during the drug discovery process. To do so we first focused on the inter-site alignment of protocols to obtain a common model of moDCs, so that we could measure the up-regulation of moDC activation markers, using either a maturation cocktail of cytokines or LPS, conditions that were then kept as positive controls for further experiments. The four therapeutic antibodies that were chosen for this study are classically used to treat multiple sclerosis and inflammatory diseases and are under evaluation for their immunogenicity potential by the ABIRISK consortium (36). First, two types of physical stress, heat and shear, were compared. As stated, the results showed different behaviors between stressed antibody materials. Although the aggregation process has been extensively studied for antibodies, only few studies showed comparison between identified monoclonal antibodies submitted to the same stress (34, 37). Our work demonstrated that infliximab and rituximab showed the highest capacity to aggregate under heat stress conditions with a fluffy visual aspect. This observation could be expected regarding the chimeric status of these antibodies, that may be less stable than natalizumab (humanized) or adalimumab (fully human). However, the propensity to aggregation also depends on the stress condition, as shear stress had less effect on aggregation for the chimeric antibodies. This suggests that other factors, as intrinsic physical and chemical properties take place in the aggregation process.

Then we assessed the discriminatory potential of the cell assays. The overall results generated by independent sites and orthogonal readouts were in correlation and support the use of these assays to investigate the intrinsic capacity of therapeutic antibodies to activate DCs, allow ranking of the tested compounds and evaluating the impact of formulation effects on responses. Indeed infliximab, that had the highest propensity to form aggregates with heat stress, induced a marked activation of moDC as measured by an increase in surface maturation marker expression (CD83 and CD86), cytokine/chemokine transcript upregulation and release (IL-1β, IL-6, IL-8, IL-12p70 and TNF-α, CCL3 and CCL4) and intracellular signaling protein phosphorylation (Syk, ERK1/2, and Akt). In contrast, natalizumab which does not aggregate under the same heat stress conditions did not induce any moDC activation in any assay read-out, whereas rituximab and adalimumab that showed a less susceptibility to heat stress could show some marker up regulation (e.g., CD80 for adalimumab, Syk and ERK for rituximab). In view of these results only heat stress (but not shear stress) aggregated infliximab induced a full maturation of moDC regarding membrane expression of activation proteins and cytokine secretion. Indeed, these two features (together with antigen presentation), are known to be necessary to induce naïve cell activation (38, 39). Both CD83 and CD86 up-regulation have been observed in studies evaluating the impact of aggregated antibodies on DCs (33–35) with the expression increase dependent on the nature and the force of the stress condition and correlate with the extent of aggregation in the antibody preparations. Regarding the cytokine release, the identified signature is in favor of a DC1 (inflammatory) phenotype, and in agreement with the one already found (35), again depending on the applied stress. In particular, a stir-stressed rituximab preparation induced more cytokine release than heat or shear stressed preparations, using the same moDC stimulation protocol and subsequent cytokine quantification (35), whereas another stir-aggregated rituximab preparation did not induce any cytokine up regulation (34). Besides, in a PBMC *in vitro* model, the early cytokines induced by stir-aggregated antibodies highlighted the predominant response of monocytes, as IL-1β, IL-6, IL-8, TNF-α, CCL2, CCL3, and CCL4 were among the most up-regulated (32, 40). Our results along with those found in the literature (32, 33, 40) tend to demonstrate that the magnitude and pattern of cellular responses correlate with the specific nature of the aggregates they interact with.

The moDC extra-cellular responses were complemented here for the first time by intracellular investigations into aggregate-induced signaling pathway activation. In the case of antibodies, Syk recruitment suggests the engagement of Fc receptors by native or aggregated forms (41). The engagement of FcγRI and FcγRIII by antibody aggregates and their impact on cytokine secretion have been demonstrated in a PBMC *in vitro* model (32). However, *in vitro* generated moDC mostly express FcγRII receptor (28), that seem to be less involved in aggregate-mediated signalization to induce cytokine release (32). We hypothesize that the Syk phosphorylation observed when moDC were treated with infliximab HSL2 could also be likely through the C-type lectin receptors. Indeed it has been described that agonists against Dectin-1 trigger Syk and ERK phosphorylation and induced DC maturation and the secretion of proinflammatory cytokines, including IL-6, TNF-α, and IL-23, but little IL-12 (42, 43). This is consistent with our cytokine data, where significant IL-6, TNF-α, and IL-12p40 transcripts were observed. While IL-12p40 is involved in both IL-12 and IL-23 production, IL-12p70 is involved in specifically IL-12 production (44). Since significant increase in IL-12p40 was observed in qPCR with limited IL-12p70 production in the MSD assay, this suggests that IL-23 is likely produced in addition to IL-12. Alternatively, it has been shown that Syk is also linked to DC-SIGN engagement, which rather modulates DC maturation through ERK and Akt activation, leading to the down regulation of cytokine production (45). Moreover, other studies showed that ERK and Akt phosphorylation modulate the effects of TLR-4 engagement by LPS, leading to a limited expression of inflammatory cytokines, including IL-12p70 (46, 47). Yet, TLR-4 was also shown to be engaged by antibody aggregates (32). In total, our results are in frame with the hypothesis that aggregates can have antibody-specific interaction with DCs through Fc receptors but may also act as danger signals interacting with many different pattern recognition receptors, that could lead to the activation of various signaling pathways.

The capacity of the different antibody aggregates to induce moDC maturation may be explained in different ways. The most evident is the propensity of the protein to aggregation, as evidenced by the comparison between infliximab and natalizumab. Indeed, a recent study of the crystal structure of infliximab revealed a reversible self-association interface in the Fab domain (48), that could favor aggregation under stress conditions. This observation could explain that in our study aggregated preparations of infliximab showed greater number of microparticles than the other aggregated antibody preparations (manuscript in preparation). Besides, it has been demonstrated that particles around 5–10 μm size were better able to activate PBMC than nano-sized particles (40). Another explanation could be related to the capacity of aggregates to interact with different receptors on moDC, as our results showed that different antibody preparations induce differential intracellular signaling pathways and subsequent cellular responses. Differential processing of aggregated antibodies has already been demonstrated, and showed that HLA-DR associated peptides were different between two different aggregated antibodies (33). The comparison of the clusters of HLA-DR presented peptides between the four antibodies tested in this study is currently under investigation (manuscript in preparation).

Several *in vitro* assays are currently in use to understand the impact of product-related factors and impurities on therapeutic antibody induced immunogenicity—reviewed in Brennan et al. (49). The impact of aggregated antibodies on the development of immune responses have been assessed using DC maturation assays (33–35), or evaluating naïve T-cell activation, either in a total PBMC culture model (32, 34, 37) or in a DC/T cell co-culture model (33, 35). Globally, all assays have shown that cellular activation is increased by aggregated preparations compared with the native products. Since tested aggregated preparations contained a greater number of particles than commercial native products, it would be of interest to determine a threshold of particle numbers that is sufficient to induce cellular activation to determine the assay sensitivity. The advantage of the DC maturation assays described here is that they may be used to compare large numbers of aggregated preparations -including different aggregation states- with the same donor, which may be interesting during early stages of BP development. The question to answer would then be whether a given form of aggregates (or a given percentage) could have the potential to induce an immune response starting with DC activation. For this purpose, the use of a single or two orthogonal read outs such as membrane activation markers and/or cytokine release would be sufficient, while the qPCR method could be used as a first screen to identify the cytokines to be quantified. Indeed, such assays could help in the selection of antibody candidates. On the other hand, the use of the signaling assay brings information to determinate the molecular mechanisms that lead to DC maturation. This is a valuable tool to gain insight into the intra-cellular pathways that are directly activated by aggregates, which are not fully understood. So, better than a screening application, this kind of assay is more dedicated to collect information to improve our understanding on DC-aggregates interaction and behavior.

In conclusion, the use of DC activation assays is useful for screening during the development phase of therapeutic antibodies for the compound's intrinsic capacity, including formulation related properties such as aggregation that may increase the risk of inducing an immune response. The extrapolation to predict the immunogenic risk of bioproducts at the clinical stage is not sufficient, as there are other influencing factors either related to patients or to the concomitant treatments (14, 50). However, it can be noted that infliximab, which has the greatest propensity to aggregate *in vitro* is one of the most immunogenic in clinical practice (6, 8). If the chimeric status of an antibody (e.g., infliximab or rituximab) can certainly be correlated to aggregation propensity and immunogenicity, this correlation seems more difficult to establish regarding humanized and fully human antibodies. Indeed adalimumab (fully human) is described as more immunogenic than natalizumab (humanized), although both antibodies are poorly susceptible to aggregation.

Nevertheless, these assays are a valuable tool to assess the cellular mechanisms that drive DC activation by aggregated proteins. Thus, we offer these optimized *in vitro* assays for mAb evaluation in particular in terms of immunogenicity driven by DC.

## Author Contributions

IT, HM, S-YT, and PB wrote the paper. YG, MN, ML, NS, SR, ZG, CZ, JL, and MY performed the assays. CZ, AK, H-WV, FA, and MP reviewed the paper.

### Conflict of Interest Statement

HM, ZG, SR, and AK are currently or were previously employed by Novartis. S-YT, JL, MY, FA, ML, PB, CZ, and H-WV are currently or were previously employed by Bayer. The remaining authors declare that the research was conducted in the absence of any commercial or financial relationships that could be construed
