# Supplementary material for: Evaluation of in vitro Assays to Assess the Modulation of Dendritic Cells Functions by Therapeutic Antibodies and Aggregates
Source: Front Immunol. 2019 Mar 28;10:601. doi: 10.3389/fimmu.2019.00601 (PMC6455063; doi:10.3389/fimmu.2019.00601)
Supplement: Supplementary file 1 [file Table_1.docx]

**INFLIXIMAB**

**Supplemental Table 1**: Protein levels of proinflammatory cytokines in moDC following treatment with native or aggregated infliximab. Immature moDC were treated for 48 h with native or stressed (SSL2 or HSL2) antibody, or maturation cocktail (MC) or LPS. Cytokine concentrations were measured in culture supernatants using MSD multiplex assay. Results are expressed as fold change over PBS control.
