# Supplementary material for: Evaluation of in vitro Assays to Assess the Modulation of Dendritic Cells Functions by Therapeutic Antibodies and Aggregates
Source: Front Immunol. 2019 Mar 28;10:601. doi: 10.3389/fimmu.2019.00601 (PMC6455063; doi:10.3389/fimmu.2019.00601)
Supplement: Supplementary file 3 [file Table_3.docx]

**A. Infliximab**

**B. Natalizumab**

**Supplemental Table 3:** Secretion of chemokines CCL3 and CCL4 secretions by moDC treated with native or aggregated infliximab (A), and natalizumab (B). Immature moDC were treated for 48 h with native or stressed (SSL2 or HSL2) antibodies, maturation cocktail (MC) or LPS. Chemokine concentrations (pg/mL) were measured in culture supernatants using a MSD multiplex assay.
