# Supplementary material for: Evaluation of in vitro Assays to Assess the Modulation of Dendritic Cells Functions by Therapeutic Antibodies and Aggregates
Source: Front Immunol. 2019 Mar 28;10:601. doi: 10.3389/fimmu.2019.00601 (PMC6455063; doi:10.3389/fimmu.2019.00601)
Supplement: Supplementary file 4 [file Image_1.pdf]

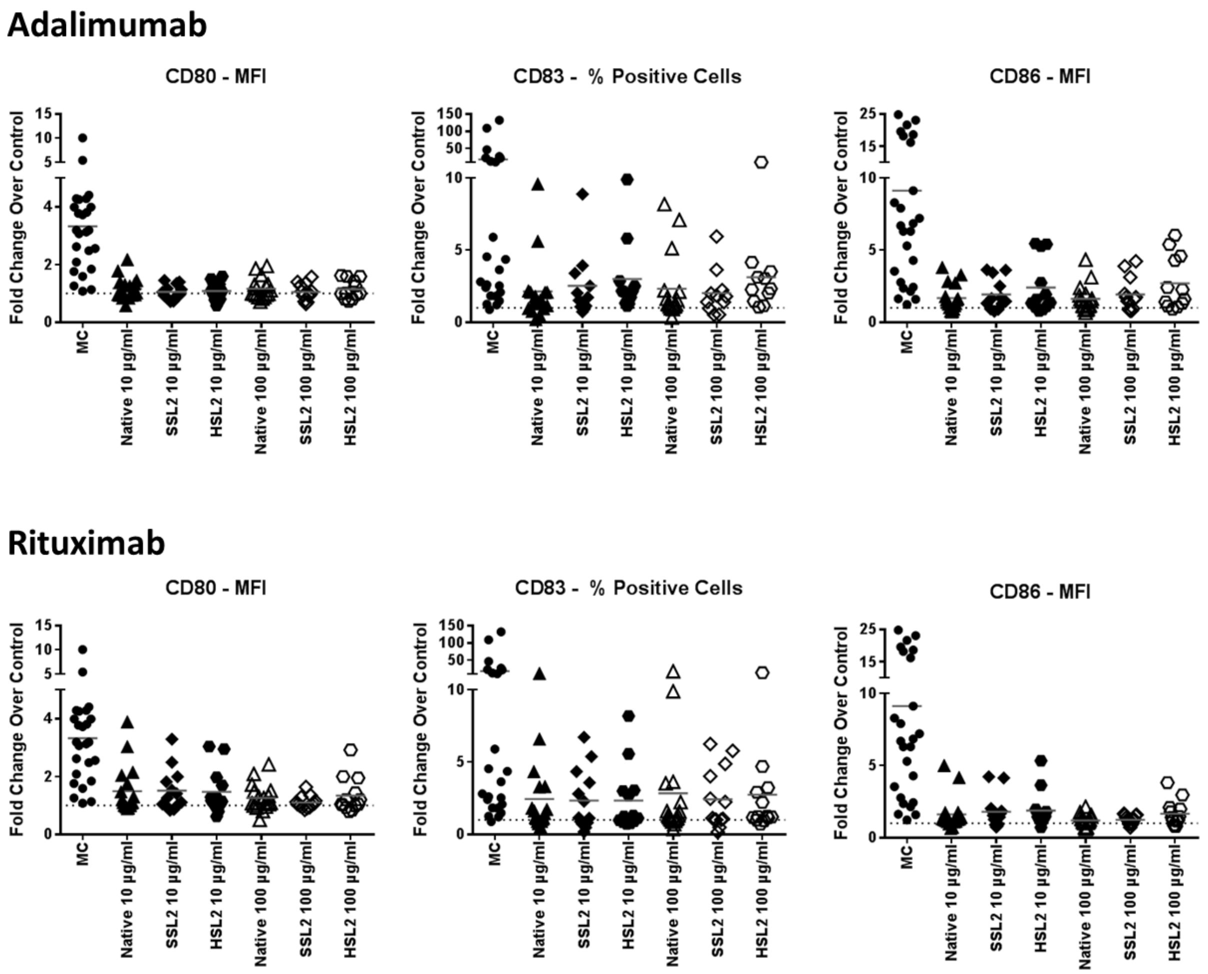

Supplemental Figure 1:

Phenotypic assessment of maturation marker expression on moDC treated with native or aggregated adalimumab (A) and rituximab (B). Immature moDC were treated for 48 h with the positive control (MC; maturation cocktail as described in the methods section) or with either native or stressed (SSL2 or HSL2) therapeutic antibodies at 10 or 100 µg/mL. Cells were collected, washed and analyzed by flow cytometry and the surface marker expression of three maturation markers (CD80, CD83 and CD86) was measured on CD11c+ moDC. Results are expressed as fold change of marker expression (either as the MFI or as the % of positive cells based on the sensitivity of responses to the positive control for each marker) compared to PBS treated cells (represented by the horizontal dotted line at 1.0). Results from 12-23 independent experiments are shown, individual points represent individual donor responses and the group mean is represented by the horizontal grey line.
