# Supplementary material for: Evaluation of in vitro Assays to Assess the Modulation of Dendritic Cells Functions by Therapeutic Antibodies and Aggregates
Source: Front Immunol. 2019 Mar 28;10:601. doi: 10.3389/fimmu.2019.00601 (PMC6455063; doi:10.3389/fimmu.2019.00601)
Supplement: Supplementary file 5 [file Image_2.pdf]

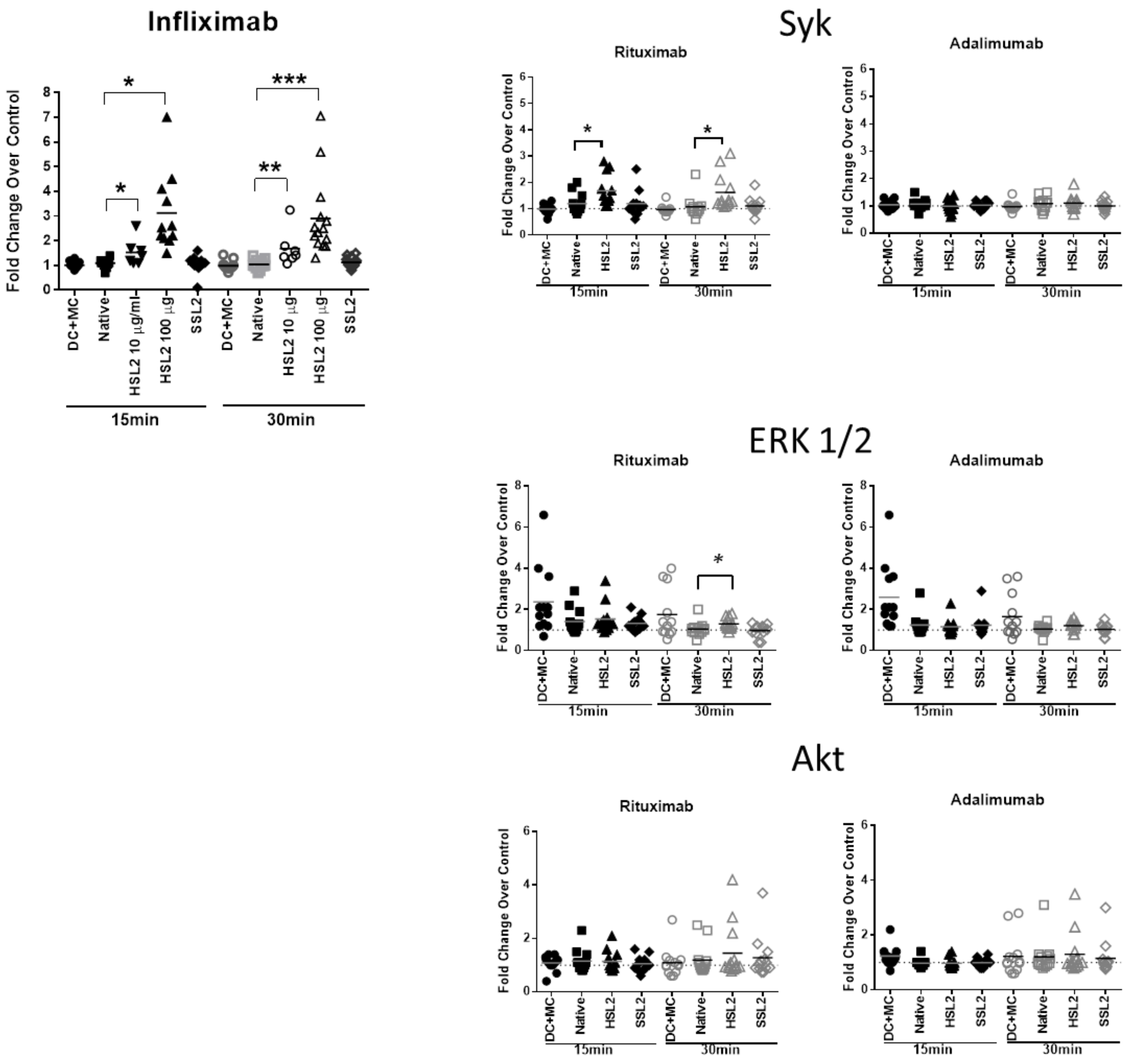

Supplemental Figure 2:

Protein phosphorylation by native or aggregated antibodies. (A) Syk phosphorylation is concentration-dependent in moDC treated by infliximab HSL2 aggregates. (B) Syk, ERK1/2 and Akt phosphorylation in moDC treated with rituximab or adalimumab and aggregates. Phosphorylation was detected following 15 and 30 min of stimulation. Results are expressed as fold change over PBS control. Results from 12 independent experiments are shown. *p<0.05; **p<0.01; ***p<0.001.
